# Detection of a New Genetic Cluster of Influenza D Virus in Italian Cattle

**DOI:** 10.3390/v11121110

**Published:** 2019-11-30

**Authors:** Chiara Chiapponi, Silvia Faccini, Alice Fusaro, Ana Moreno, Alice Prosperi, Marianna Merenda, Laura Baioni, Valentina Gabbi, Carlo Rosignoli, Giovanni L. Alborali, Lara Cavicchio, Isabella Monne, Camilla Torreggiani, Andrea Luppi, Emanuela Foni

**Affiliations:** 1 Istituto Zooprofilattico Sperimentale della Lombardia ed Emilia-Romagna, 25124 Brescia, Italy; silvia.faccini@izsler.it (S.F.); anamaria.morenomartin@izsler.it (A.M.); alice.prosperi@izsler.it (A.P.); marianna.merenda@izsler.it (M.M.); laura.baioni@izsler.it (L.B.); valentina.gabbi1@studenti.unipr.it (V.G.); carlo.rosignoli@izsler.it (C.R.); giovanni.alborali@izsler.it (G.L.A.); andrea.luppi@izsler.it (A.L.); emanuelafoni0956@gmail.com (E.F.); 2 Istituto Zooprofilattico Sperimentale delle Venezie, 35121 Padua, Italy; afusaro@izsvenezie.it (A.F.); LCavicchio@izsvenezie.it (L.C.); imonne@izsvenezie.it (I.M.)

**Keywords:** influenza D virus, cattle, phylogenetic analysis, Italy

## Abstract

Influenza D virus (IDV) has been increasingly reported all over the world. Cattle are considered the major viral reservoir. Based on the hemagglutinin-esterase (HEF) gene, three main genetic and antigenic clusters have been identified: D/OK distributed worldwide, D/660 detected only in the USA and D/Japan in Japan. Up to 2017, all the Italian IDV isolates belonged to the D/OK genetic cluster. From January 2018 to May 2019, we performed virological surveillance for IDV from respiratory outbreaks in 725 bovine farms in Northern Italy by RT-PCR. Seventy-four farms were positive for IDV. A full or partial genome sequence was obtained from 29 samples. Unexpectedly, a phylogenetic analysis of the HEF gene showed the presence of 12 strains belonging to the D/660 cluster, previously unreported in Europe. The earliest D/660 strain was collected in March 2018 from cattle imported from France. Moreover, we detected one viral strain with a reassortant genetic pattern (PB2, PB1, P42, HEF and NP segments in the D/660 cluster, whilst P3 and NS segments in the D/OK cluster). These results confirm the circulation of IDV in the Italian cattle population and highlight the need to monitor the development of the spreading of this influenza virus in order to get more information about the epidemiology and the ecology of IDV viruses.

## 1. Introduction

The circulation of influenza D virus (IDV) has been detected in a wide range of animal species and has been increasingly reported all over the world since its discovery in 2011 [1,2]. Cattle are considered the major viral reservoir. Bovine respiratory disease (BRD) is a major health problem of respiratory system occurring worldwide in both dairy and feedlot cattle. Viral agents may produce a clinical syndrome consistent with BRD and their involvement is generally considered as antecedent to, or concurrent with, bacterial infection. Viruses that are commonly implicated include bovine herpesvirus-1 (BHV-1), bovine viral diarrhea virus (BVDV), bovine respiratory syncytial virus (BRSV) and parainfluenza 3 virus (PI3V) [3]. Cattle are considered the major IDV reservoir but understanding the possible role of IDV in bovine respiratory disease (BRD) is one of the main focuses of research studies concerning this virus [4,5]. BRD is a complex pathology causing economic losses and it is linked with microbial pathogens, host, environmental and animal management factors. IDV is an emerging pathogen and it has been found to be associated with BRD in metagenomic studies [6,7,8] but its pathogenic role is unclear. Cattle experimentally infected by IDV by itself showed mild to moderate respiratory disease [4,9] and IDV-positive samples are reported from both cattle manifesting clinical signs associated with BRD and from healthy animals [10,11].

Serological studies in human population showed low positivity and the zoonotic potential of this influenza virus still remains controversial [12].

Based on the hemagglutinin-esterase (HEF) gene, at least three main genetic and antigenic clusters have been identified: D/OK distributed worldwide, D/660 detected only in the USA, and D/Japan in Japan [2]. Since 2014, the co-circulation of two distinct genetically and antigenically lineages have been reported in the USA. Reassortment events have been evidenced between the two viral lineages and at least seven genotypes have been described with antigenic differences in good agreement with HEF phylogeny [13]. In Europe, IDV viruses have been detected and sequenced in France, Italy [14,15,16], Republic of Ireland and Northern Ireland (UK) [14,15,16]. D/OK is the most reported lineage circulating in Europe, even though two strains clustering separately have been reported in France and Ireland (D/bovine/France/2986/2012 and D/bovine/Ireland/07780/2014) [10,17]. In particular, since 2014, we have detected in Italy the circulation of a single genetic group within the D/OK lineage in swine and cattle population [15,16].

Here, we described the first detection in Europe of the D/660 lineage and showed the co-circulation of distinct IDV lineages, as well as of a novel reassortant virus among cattle farms in Northern Italy.

## 2. Materials and Methods

### 2.1. Virological Screening

The IDV screening was performed from 696 respiratory outbreaks in 725 bovine farms in Northern Italy. From January 2018 to May 2019, 936 samples (664 nasal swabs, 250 lung tissues and 22 bronchoalveolar fluids) were collected by field veterinarians for diagnostic purposes during bovine respiratory outbreaks in the Italian regions of Piemonte, Lombardia, Emilia Romagna, Veneto, Trentino Alto Adige and Friuli Venezia Giulia. The specimens were refrigerated at 4 °C prior to analysis. RNA extraction was performed generally within 48 h from sampling but if tests were delayed for more than 2 days, specimens were frozen at −70 °C. Viral RNA was extracted from clinical samples using One-For-All Vet Kit (Qiagen, Hilden, Germany) according to the manufacturer’s instructions and RT-PCR was performed targeting IDV PB1 gene [18]. PCR was performed by using QIAGEN Quantifast Probe RT-PCR kit+IC with IDV F (5′TGGATGGAGAGTGCTGCTTC′), IDV R (3′GCCAATGCTTCCTCCCTGTA3′) and IDV Probe (FAM-CATGTTAAACATTCCCATCAGCATTCCT −BHQ1). Retrotranscription step was at 50 °C for 20 min followed by 95 °C for 5 min; PCR reaction was performed with 45 cycles of 15 s at 94 °C, 60 s at 60 °C followed by fluorescence detection. The analytical cutoff for positivity was established at Ct value of 38.

### 2.2. Virus Isolation

Samples positive by Real-Time RT-PCR were tested for virus isolation in human rectal tumor cells (HRT-18G) (ATCC, Manassas, VA, USA) as described by Ferguson et al. [19]. Following infection, incubation was prolonged up to 5 days, in the absence of cytopathic effect. Two serial passages were performed for each sample. Confirmation of viral replication was performed using hemagglutination test and RT-PCR test at each passage. Next generation sequencing (NGS) on Miseq instrument (Illumina, Madison, WI, USA) was conducted from IDV isolates or, if isolation was not possible, from IDV PCR-positive clinical samples [16].

*Genetic analysis.* Viral RNA was extracted from cell cultures or clinical samples using the One-For-All Vet Kit (Qiagen, Hilden, Germany) according to the manufacturer’s instructions. RT-PCR of all seven genome segments was performed as previously described [20], using SuperScript^®^ III One-Step RT-PCR System with Platinum^®^ Taq High Fidelity (Thermo Fisher Scientific, Waltham, MA, USA). RT-PCR products were purified with NucleoSpin^®^ Gel and PCR Clean-up (Macherey-Nagel, Düren, Germany). DNA libraries were made with NEXTERA-XT kit (Illumina Inc., San Diego, CA, USA) according to manufacturer’s instructions. Libraries were sequenced on a MiSeq Instrument (Illumina Inc., San Diego, CA, USA) using a Miseq Reagent Kit v2 in a 250-cycle paired-end run. Illumina reads were assembled by CLC Genomic Workbench v. 11 (Qiagen, Hilden, Germany). Gene sequences from Italian IDVs and all reference IDV sequences retrieved from Genbank were aligned with ClustalW using MEGAX [21]. Partial gene sequences were included in the analyses and the alignments were not full length. Phylogenetic trees of the individual segments (PB2 2314 nt, PB1 818 nt, P3 2133 nt, HE 635 nt, P42 1151 nt, NS 817 nt and NP 1656 nt) were inferred with the maximum likelihood (ML) method implemented in IQ-TREE package 1.6.11 [22]. The robustness of the ML trees was statically evaluated by bootstrap analysis with 1000 bootstrap replicates.

## 3. Results

**Figure 1:**
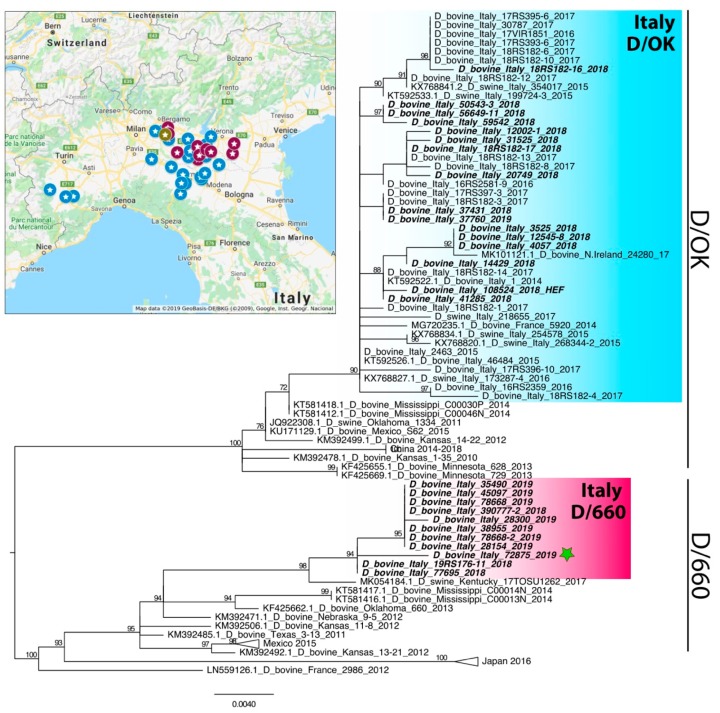
Map showing the collection site of the influenza D virus (IDV) sequenced in this study. Blue: strains clustering with D/OK, purple: strains clustering with D/660, green: reassortant strain. Phylogenetic tree of hemagglutinin-esterase (HEF) (635 nt) gene. Sequences are listed by host, country, strain name and collection year. Scale bars indicate nucleotide substitutions per site. The viruses analyzed in this study are in bold italic. The reassortant strain D/bovine/Italy/72875/2019 is marked by a green star. The groups Italy-D/OK and Italy-D/660 are highlighted in blue and purple, respectively.

**Figure 2:**
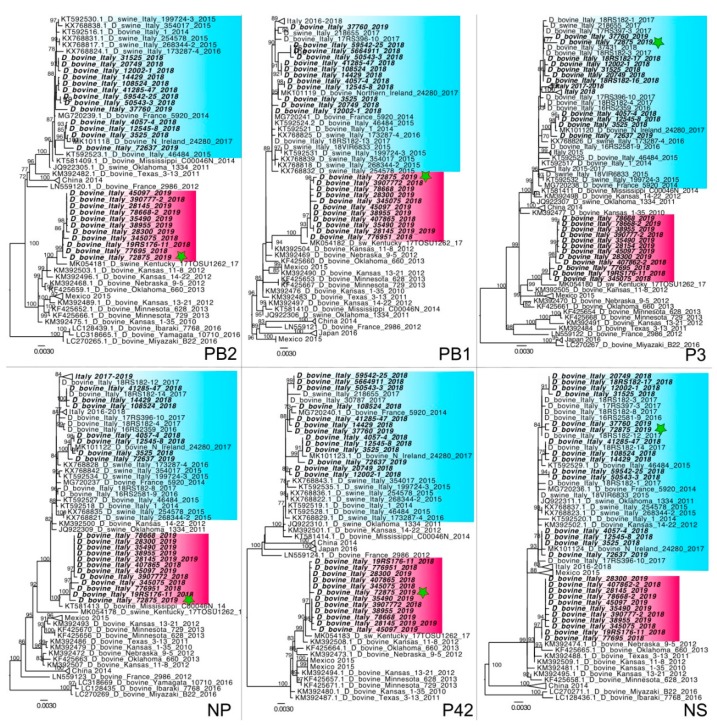
Phylogenetic trees of internal genes PB2 (2314 nt), PB1 (818 nt), P3 (2133 nt), P42 (1151 nt), NS (817 nt) and NP (1656 nt). Sequences are listed by host, country, strain name and collection year. Scale bars indicate nucleotide substitutions per site. The viruses analyzed in this study are in bold italic. The reassortant strain D/bovine/Italy/72875/2019 is marked by a green star. The groups Italy-D/OK and Italy-D/660 are highlighted in blue and purple, respectively.

Ninety-two nasal swabs and seven lung tissues from 74 farms were positive for IDV, of which 43 farms out of 585 (7.3%) in 2018 and 31 farms out of 140 (22%) in 2019. As reported above, the full genome sequencing was performed on positive samples and partial or full-length sequences were obtained from 29 samples by NGS (18 from isolated viruses and 11 from clinical samples) (Table 1) (Genbank accession numbers MN173611-MN173617, MN165175-MN165257, MN123862-MN123959). The phylogenetic analysis of the HEF sequences showed that 17 viruses fell within the previously reported D/OK lineage. Unexpectedly, the remaining 12 strains belonged to the D/660 cluster, previously unreported in Europe (Figure 1). Analyses of the remaining gene segments confirmed the co-circulation in Italy of two distinct clusters, namely Italy-D/660 and Italy-D/OK (Figure 2). Moreover, in a dairy farm, we detected one viral strain, D/bovine/Italy/72875/2019, with a reassortant genetic pattern. Indeed, the analysis of its partial genome sequence obtained directly from the clinical sample showed that PB2, PB1, NP, P42 and HEF segments fell in the Italy-D/660 group, while P3 and NS segments belonged to the Italy-D/OK group (Figure 1 and Figure 2). Investigations about both the earliest D/660 strains, D/bovine/Italy/77695/2018 and D/bovine/Italy/19RS176-11/2018, showed that they were detected from samples collected in beef-producing farms in March 2018 from cattle imported from France. In particular, the D/bovine/Italy/19RS176-11/2018 positive cattle consisted of a one-year old bovine introduced in Italy from France in March 2018, which died with respiratory disease 10 days after the introduction. All the remaining D/660 strains were detected from November 2018 to March 2019 from six dairy and four meat farms. At farm #11, in particular, we detected the circulation of the two lineages in two close samplings, performed on 29 January 2019 and 11 February 2019 (Table 1). The new IDV lineage was detected in 26.3% of the analyzed viruses in 2018 (5/19) and in 70% of the sequenced samples in 2019 (7/10) (Table 1).

## 4. Discussion

Cattle are considered the major reservoir of IDV and the role of this recently discovered influenza virus in BRD still remains controversial [23,24]. We performed a virological screening for IDV from samples collected from cattle belonging to farms in Northern Italy with respiratory symptoms. The genome characterization of the IDVs sequenced showed the co-circulation of two genetic lineages in the Italian cattle population.

All the IDV strains sequenced came from regions with high density of cattle farms where we previously demonstrated an IDV serological prevalence of 92.7% (lineage C/OK) [25].

Since the previous screening performed in Italian farms did not detect any virus of D/660 lineage [15,16,25] and since the first detected strains came out from animals imported from France and introduced in Italy just before the viral detection, it is possible to suppose that the introduction and spread of this IDV lineage in Italy is recent. In 2018, Italy imported from France 399,585 tons of live bovine animals, representing 89.2% of the imported Italian cattle [26]. However, given the lack of sequences from Europe belonging to the D/660 lineage, we cannot rule out other sources of the virus, nor can we exclude an undetected circulation of the strain in France or in the Italian territory. The positivity of dairy farms shows the spread of this virus also among Italian farms that do not import animals from other countries. Moreover, the proximity of farms infected by different lineages (Figure 1) and the demonstration of the co-circulation of the two lineages in one farm (#11) explain the presence of reassortant strains. As has already occurred in the USA [13], we can expect the detection of different reassortants in the future. Since, in 2019, more D/660 strains circulated compared to D/OK strains, a shift in time of the genotypes could be taking place. Further virological and serological studies will be helpful to elucidate and to confirm the data described herein, moreover, the circulation of this new lineage should be investigated in the swine population where this virus has been detected in Italy [15]. Our findings reveal an ongoing situation and highlight the need to continue monitoring the spread and evolution of this emerging influenza virus in order to better understand its fine-scale transmission dynamics in the European and Italian territory. The knowledge of the influenza viral circulation in a territory allow for prompt identification of the emergence of new reassortant strains with unpredictable virological properties. Given the economic impact of BRD in the cattle industry and the potential contribution of IDV to the disease [6,7,8,24], continuous surveillance is required to characterize its evolution.

## Figures and Tables

**Table 1:** IDV-positive samples analyzed in this study. The source and collection date are reported. The farm of origin of each strain is identified by number and the lineage attribution is based on the HEF segment. The availability of the isolated virus is reported in the last column. The viruses belonging to a lineage different from D/OK are in the grey rows. * The partial HEF sequence is not included in the phylogenetic tree.

Strain	Date of Sampling	Production Type	Lineage	Source	ID Farm	Isolated Virus
D/bovine/Italy/3525/2018	04/01/2018	beef	D/OK	nasal swab	13	yes
D/bovine/Italy/4057/2018	05/01/2018	beef	D/OK	nasal swab	3	yes
D/bovine/Italy/12002/2018	12/01/2018	dairy	D/OK	nasal swab	20	yes
D/bovine/Italy/12545/2018	12/01/2018	dairy	D/OK	nasal swab	24	yes
D/bovine/Italy/14429/2018	16/01/2018	dairy	D/OK	lung	7	yes
D/bovine/Italy/18RS182-16/2018	19/01/2018	beef	D/OK	nasal swab	26	no
D/bovine/Italy/20749/2018	20/01/2018	beef	D/OK	nasal swab	15	yes
D/bovine/Italy/31525/2018	31/01/2018	dairy	D/OK	nasal swab	18	yes
D/bovine/Italy/37431/2018 *	07/02/2018	beef	D/OK	nasal swab	16	yes
D/bovine/Italy/41285/2018	10/02/2018	dairy	D/OK	nasal swab	24	yes
D/bovine/Italy/50545/2018	21/02/2018	dairy	D/OK	nasal swab	4	yes
D/bovine/Italy/56649/2018	27/02/2018	dairy	D/OK	nasal swab	5	yes
D/bovine/Italy/59542/2018	01/03/2018	dairy	D/OK	nasal swab	27	yes
D/bovine/Italy/77695/2018	20/03/2018	beef	D/660	nasal swab	8	no
D/bovine/Italy/19RS176-11/2018	29/03/2018	beef	D/660	nasal swab	25	no
D/bovine/Italy/108524/2018	18/04/2018	beef	D/OK	nasal swab	6	yes
D/bovine/Italy/345075/2018	14/11/2018	beef	D/660	nasal swab	23	yes
D/bovine/Italy/390777/2018	13/12/2018	dairy	D/660	nasal swab	10	yes
D/bovine/Italy/407865/2018 *	27/12/2018	dairy	D/660	nasal swab	1	no
D/bovine/Italy/28300/2019	23/01/2019	beef	D/660	nasal swab	12	no
D/bovine/Italy/28145/2019	23/01/2019	dairy	D/660	nasal swab	9	no
D/bovine/Italy/35490/2019	29/01/2019	dairy	D/660	nasal swab	11	no
D/bovine/Italy/37760/2019	30/01/2019	beef	D/OK	nasal swab	22	yes
D/bovine/Italy/38955/2019	31/01/2019	dairy	D/660	nasal swab	14	no
D/bovine/Italy/45097/2019	06/02/2019	beef	D/660	nasal swab	21	yes
D/bovine/Italy/50660/2019	11/02/2019	dairy	D/OK	nasal swab	11	no
D/bovine/Italy/72875/2019	27/02/2019	dairy	reassortant	nasal swab	19	no
D/bovine/Italy/72637/2019	27/02/2019	dairy	D/OK	nasal swab	17	no
D/bovine/Italy/78668/2019	05/03/2019	beef	D/660	nasal swab	2	yes
